# Barriers and facilitators to seeking and accessing mental health support in primary care and the community among female migrants in Europe: a “feminisms” systematic review

**DOI:** 10.1186/s12939-023-01990-8

**Published:** 2023-09-26

**Authors:** Patrick Nyikavaranda, Marija Pantelic, Christina J Jones, Priyamvada Paudyal, Alice Tunks, Carrie D Llewellyn

**Affiliations:** 1 Department of Primary Care & Public Health, Brighton & Sussex Medical School, University of Sussex, Watson Building, Room 104, Falmer, Brighton, East Sussex, BN1 9PH UK; 2 School of Psychology, Faculty of Health and Medical Sciences, University of Surrey, Guildford, GU2 7XH Surrey UK; 3 Institute for Global Health and Wellbeing School of Medicine, Keele University, Keele, Staffordshire, ST5 5GB UK

**Keywords:** Female migrants, Refugees, Asylum seekers, Mental Health, Access, Primary care

## Abstract

**Background:**

Recent years have seen record levels of migration to Europe. Female migrants are at heightened risk of developing mental health disorders, yet they face barriers to accessing mental health services in their host countries. This systematic review aims to summarise the barriers and facilitators to accessing mental health support for female migrants in Europe.

**Methods:**

The review follows PRISMA guidelines, and the protocol was pre-published on PROSPERO. Six electronic databases were searched: CINAHL, Global Health Database, Medline, PsycARTICLES, PsycINFO and Web of Science. Thematic analysis was undertaken on the identified studies. A feminist quality appraisal tool was applied.

**Results:**

Eight qualitative, six quantitative and five mixed methods studies were identified. Barriers included a lack of information, stigma, religious and cultural practices and beliefs, and a lack of consideration of gender-specific needs within the health system. Gender-sensitive services, supportive general practitioners and religious leaders facilitated access.

**Conclusions:**

The design of mental health research, services, policies, and commissioning of support for migrants must consider female migrant needs. Mental health support services must be culturally aware and gender sensitive.

**Registration:**

The review protocol was registered on the International Prospective Register of Systematic Reviews (PROSPERO, registration number CRD42021235571.

**Supplementary Information:**

The online version contains supplementary material available at 10.1186/s12939-023-01990-8.

## Introduction

As of 2020, over half (51.6%) of all international migrants to Europe were female [1]. In recent years there have been record levels of migration to Europe of individuals born outside of Europe; reasons for migration include work, family reunification and protection seeking [2–6]. In January 2021, over 447.2 million inhabitants were living in the European Union (EU) of which 23.7 million were non-EU citizens [7]. Refugees accounted for only 0.6% of the total EU population. In contrast, refugees hosted by Lebanon account for 12% of the total population. Asia, Africa, and the Middle East were areas of origin for the most first-time asylum applicants to the EU, with Syria, Afghanistan, Iraq, Pakistan, and Turkey accounting for the most nationalities. Germany, France, Spain, Italy, and Austria were the countries with the most first-time applications for asylum [8]. The so-called ‘Refugee Crisis’ in Europe saw 40% of land arrivals to Europe identified as women and children [9]. Female migration may be complicated by gender-specific challenges which are likely to continue or be exacerbated whilst in transit, upon arrival and during integration within their host countries [10–13]. Within European countries, female migrants face challenges which may include unsafe housing and predatory caseworkers for those seeking asylum, and harassment in the workplace because of their migrant background and gender [13, 14]. In the United Kingdom (UK), one in five females seeking asylum has experienced gender-based abuse [15]. Disclosure of violence, victimisation and mental health concerns in this population remains a challenge due to a host of factors, some of which may include a lack of awareness of support mechanisms, threats, shame, guilt, and fears of being deported should they disclose the abuse or any additional challenges [16–18].

Evidence suggests that females in newcomer populations regardless of the migrant description, including labour migrants, those moving for family reasons, refugees, and asylum seekers, are at a higher risk of diagnosable mental health-related illnesses compared to male migrants and the general population [19–21]. Female migrant populations, particularly forced migrants, and those in the perinatal phase, are disproportionately affected by post-traumatic stress disorder (PTSD) over the course of their lives [22, 23]. Risk factors for female migrant populations include trauma, social isolation, discrimination, and financial hardship [24].

Besides PTSD, female migrants, particularly refugee women, are disproportionately at risk of prenatal depression. One study suggests that they are 37.5% more likely to experience perinatal depression [24]. A global study estimated that the prevalence of perinatal depressive disorders among female migrants is between 19% and 31%, which is significantly higher than the prevalence of depression (12–17%) in the general population of perinatal females [25]. The burden of perinatal mental illness (depression, anxiety, and post-traumatic stress disorder) is disproportionately high among female migrants. One in four female migrants who are pregnant or postpartum experience perinatal depression, sadly, one in five experiences perinatal anxiety, and one in 11 experiences perinatal PTSD [25]. However, the burden of perinatal mental illness appears to be higher among forced migrants than economic migrants.

Some migrant populations have unique challenges, for example, their lack of legal status may limit access to appropriate support such as in the case of undocumented and asylum-seeking mothers who fear deportation should they seek support [26, 27]. Studies have reported differences in mental health outcomes amongst different migrant populations [28, 29]. However, consideration for the similarities in the experience of migrant populations in accessing support from a gendered perspective has not always been so. There are near-similar experiences from a gendered perspective of the different female migrant populations in access and treatment compared to male counterparts and the general population [30–33]. Rather than migrant status differences, gender issues need to be brought to the fore as most studies show gender is a greater factor over any others in terms of outcomes e.g., mental health, cancer screening, and smoking cessation [30, 34–36]. Research runs the risk of glossing over gender and sex differences and similarities, like how indigenous female migrants crossing into the United States of America are effectively invisible due to being classed as Latinx or Indians [37]. Referral to secondary mental health services and service utilisation amongst female migrants is low, and one of the reasons for this might be the lack of and inadequacy of support structures for female migrants [34, 38]. Good coordination and provision of adequate services have increased positive outcomes of mental health disorders [39]. Furthermore, female migrants have shown resilience, coping mechanisms, and posttraumatic growth after facing challenges pre- and post-migration [40–42]. The definition of who is a ‘migrant’ is problematic as there is no formal legal definition, resulting in differing definitions depending on the law, research, and public debate. The United Nations defines an international migrant as ‘someone who changes his or her country of usual residence’ [43]. The lack of a clear definition has consequences on how people access health support within primary and secondary care [44, 45]. Studies have investigated migration in the context of grouping second-generation young people, labour migrants, asylum seekers and refugees under an umbrella term of ‘migrant groups’ [46, 47]. Distinctions between economic/labour migrants and asylum seekers in the member states of the EU may not always be clear, though international law accords them with different levels of protection and assistance [6, 48, 49]. Individuals coming from the same country may also have different migrant group statuses, for example, individuals from the former USSR countries who ended up being economic migrants, refugees or moving for family reunification in Switzerland fell under the ‘migrant’ umbrella [50]. Migrant workers, refugees and asylum-seeking individuals experience similar barriers to accessing support for their mental health in host countries [51–54]. The added layer of having a label of ‘’migrant’’ or ‘’immigrant’’ for a population that is traditionally faced with more inequalities compared to host populations demonstrates the intersectional, yet often complicated nature of female migrants placed in different categories.

In the context of inequalities faced by females and the different groupings of female migrants, it is, therefore, necessary to investigate the experiences and perceptions of migrants identified as female, accounting for their migration status but not discounting the one unifying label of ‘’female’’. For the current review, we use the terms ‘migrant’ and ‘newcomer populations’ as a grouping of these different categories, however, we acknowledge differences in experiences and status and how these may impact mental health access throughout the review.

Migration research largely focuses on men [55], which has led to services being designed with male migrants in mind and little input from and consideration for female migrant populations. Indeed, whilst male migrants utilise mental health services less than the general population, female migrants make disproportionately less use of these services compared to the male migrant population [19]. A male narrative-dominated review of perceptions and experiences of access does not reflect female migrant views in understanding the collective experiences and perceptions of females with a migrant label regardless of migrant status. Inequalities are found among class, race, sexuality, gender, and power. Within these different inequalities, the group traditionally labelled as ‘’female’’ has faced even more inequalities within these inequalities [31–33, 56]. The present review acknowledges the controversies within feminist theories and gender studies [57]. Additionally, it challenges the stereotypical views of migrant males being ‘’a risk’’ and female migrants being ‘’at risk’’ as widely reported by the press which feeds into negative or stereotypical public perceptions of migrants [56]. Therefore, the present review takes a generalist approach to discussing issues affecting females including gender and sex, and the intersection between race, class, gender, and identity. Conclusions of female migrant issues have formed largely due to historic, colonial, and often racialised thinking without the need to understand the direct views and experiences of female migrants who may have more than just ‘’migrant’’ as a label as they can be female and have different sexualities and religions [58–61].

Through a feminisms lens, the review seeks to highlight the traditional inequalities faced by women and girls from newcomer populations. It seeks to add to the attempts within feminist theories of defining, establishing, and achieving personal, social, political, and economic rights for women and girls through the focus on gender as a system influenced by migration and intersectionality [62]. Incorporating transnational and intersectional feminisms and decolonial perspectives, in the words of Dr Nof Nasser-Eddin, is to *“look at the system or systems of oppression that make our struggles much more unified*” [63]. This means investigating what unifies the different female migrant populations as opposed to categorising them by migrant status, thus weakening the need to address their struggles, for “sisterhood is global” [64].

This review aims to address important gaps in the literature by identifying the barriers and facilitators to help-seeking and accessing mental health (MH) support for female migrants. The review employs a feminisms lens to identify issues relating to constructs of feminist theories raised within these studies and how these influence research, policy, provision of services and access routes to support the mental health of female migrants [55, 65–68].

## Methods

This paper follows the Preferred Reporting Items for Systematic Reviews and Meta-analyses (PRISMA) [69]. The review protocol was registered on the International Prospective Register of Systematic Reviews (PROSPERO, registration number CRD42021235571).

### Eligibility

Studies were considered eligible for the review if they included (i) female participants who identified as migrants, asylum seekers or refugees, and data could be extrapolated pertaining to female experiences where both genders were included, (ii) focused on any common mental health conditions (CMHCs) (e.g., PTSD, depression, and anxiety disorders) and (iii) perceived barriers and facilitators to accessing formal or informal mental health support. Formal mental health support in the context of this review is characterised by scheduled appointments, time constraints, and professional expertise. It is provided by trained professionals, such as doctors, psychologists, social workers, and psychiatrists. Informal help, on the other hand, is characterised by emotional closeness, companionship, and reciprocity. It is often provided by friends, relatives, and religious and community support groups. Similar definitions have been used in other studies [70–72]. Primary care is the first point of contact for healthcare, providing comprehensive, integrated, person-centred services to meet the majority of personal health needs [73]. Access to community and primary care support is mostly for CMHCs [74, 75], hence, the review focuses on CMHCs.

No limitation was applied to participants’ age, date of study publication or design. Within Europe, 27 countries comprise the European Union (EU) and the EU single market countries outside of the EU: Iceland, Liechtenstein, Norway, Switzerland, and the UK were included [76]. Europe was the focus of the review owing to many factors which include more similar policies and practices for healthcare delivery, and more countries within the European Economic Area allowing free movement of individuals between borders. Additionally, the review focused on Europe as it is a popular destination for people from high, low-and-middle- income settings from different geographic regions of the globe, which in turn confers a unique challenge in providing health services.

The settings considered for the study were primary care, which included general practitioners (GPs) and community care, including informal and formal support. Example subject index terms included: female migrants, access, mental health, primary care, community health services, and Europe.

### Search strategy

To identify the relevant articles, the team used the SPIDER search tool, which stands for Sample, Phenomenon of Interest, Design, Evaluation, and Research Type. SPIDER, designed by Alison Cooke and her colleagues is specifically designed to identify relevant qualitative and mixed-method studies [77]. Index terms were combined with Boolean operators. See Table 1.

**Table 1:** SPIDER Search Strategy

SPIDER	Search Terms
Sample	refugee* OR asylum OR migrant* OR immigrant* OR emigrant* OR displac* person* OR displac* population* OR migrat* OR (minority ethnic groups) OR Exile*
Phenomenon of Interest	AND (Mental illness) OR (mental disorder) OR (common mental health problems) OR (mood disorder) OR (emotional problems) OR trauma OR distress OR anxi* OR depress* OR stress OR (common mental health symptoms) OR (major depressive disorder) OR (mental health problem) OR wellbeing OR well-being OR (low mood) OR dysthymia OR phobia* OR (panic disorder) OR (post-traumatic disorder) OR (posttraumatic stress disorder) OR PTSD AND (Psychological Therapy) OR IAPT OR (Improving Access to Psychological Therapies) OR GP OR (general practice*) OR (primary care) OR (mental health services) OR (Psychological Treatment) OR psychothera* OR counselling OR CBT OR (cognitive behavioural therapy) OR psych* OR (community intervention) OR (peer support) OR (community engagement) OR CMHT OR (Community Mental Health Team) or (Community support) AND Access* OR Exclusion OR (Low representation) OR Non-attendance OR Help-seek* OR (Failure to attend) OR (service utilisation) OR (treatment participation) OR (treatment engagement) OR (unmet need) OR (service engagement) OR attend* barriers OR (treatment seeking) OR non-referral OR self-referral OR (support seek*) OR Uptake OR (pathways to care) OR (Health Service Access*) OR seek* help OR (seek* support) OR (seek* treatment) OR Stigma
Design	
Evaluation	Barrier* OR Facilitator*
Research Type	Qualitative or Quantitative

### Study screening and selection

Reviewers PN and AT searched six electronic databases from the date of database inception to 10 March 2021. PN and AT independently screened titles and abstracts. All full texts were independently evaluated by PN and AT for inclusion, with the full agreed list of full text sent to CDL, CJJ, PP and MP to assess suitability for inclusion. Updated searches were conducted in May 2022 by PN and discussed with the review team for inclusion. The databases searched were: CINAHL, Global Health Database, Medline PsycARTICLES, PsycINFO and Web of Science. A references search to related reviews and a ‘reverse citation’ exercise on Google Scholar were conducted to see whether the included articles had subsequently been cited.

### Quality assessment and presentation

The Mixed Methods Appraisal Tool (MMAT) – Version 2018 was used to critically appraise the reporting of the included studies. The MMAT was chosen because it is a multi-purpose tool that can be used for qualitative research, randomized controlled trials, non-randomized studies, quantitative descriptive studies, and mixed methods studies [78]. The MMAT is used to assess the quality of the reporting of a study, rather than the quality of the study itself. Therefore, no paper was rejected based solely on its quality assessment.

Two independent reviewers (PN and AT) undertook an appraisal of all included papers using the MMAT. The reviewers’ scores were then compared, and any discrepancies were discussed. This process ensured that the quality assessment was reliable and consistent.

The Feminist Quality Appraisal Tool by Morgan and colleagues (2017) was selected for appraisal from a feminist perspective as it draws upon feminisms including radical, constructionist, and intersectional perspectives [79]. Furthermore, as it investigates constructs of gender, it also seeks to address health inequalities based on gender in this instance, as the exclusion of female knowledge and experiences in a male-dominated knowledge and experience world may lead to the very inequalities being sought to be eradicated [68]. Definitions have been abridged from the study by Morgan and colleagues (2017). This is a subjective measurement of study quality; however, it benefits the review process as it is not constricted to choosing between implicit and explicit components of studies. PN and AT independently assessed the included studies in discussion with the rest of the review team.

### Analysis

The text of included articles was imported into QSR International’s NVivo 12 qualitative data analysis software [80]. For any included quantitative studies, data were extracted independently by PN through summaries of key outcomes and researcher interpretations of the data. Results were checked by CDL, CJJ, MP and PP. Inductive thematic analysis was used to present prominent themes from the qualitative studies [81]. Through data familiarisation of the findings and author interpretations, these generated codes were synthesised to prominent themes.

### Lived experience contribution to the review

A co-production group of female migrants and professionals who support female migrants met to discuss the issues facing female migrants. This discussion led to the development of an initial analysis framework for research on female migrant mental health. The group has continued to contribute to subsequent research on this topic. The co-production have provided a commentary on the review (Supplementary File 1).

## Results

**Fig. 1:**
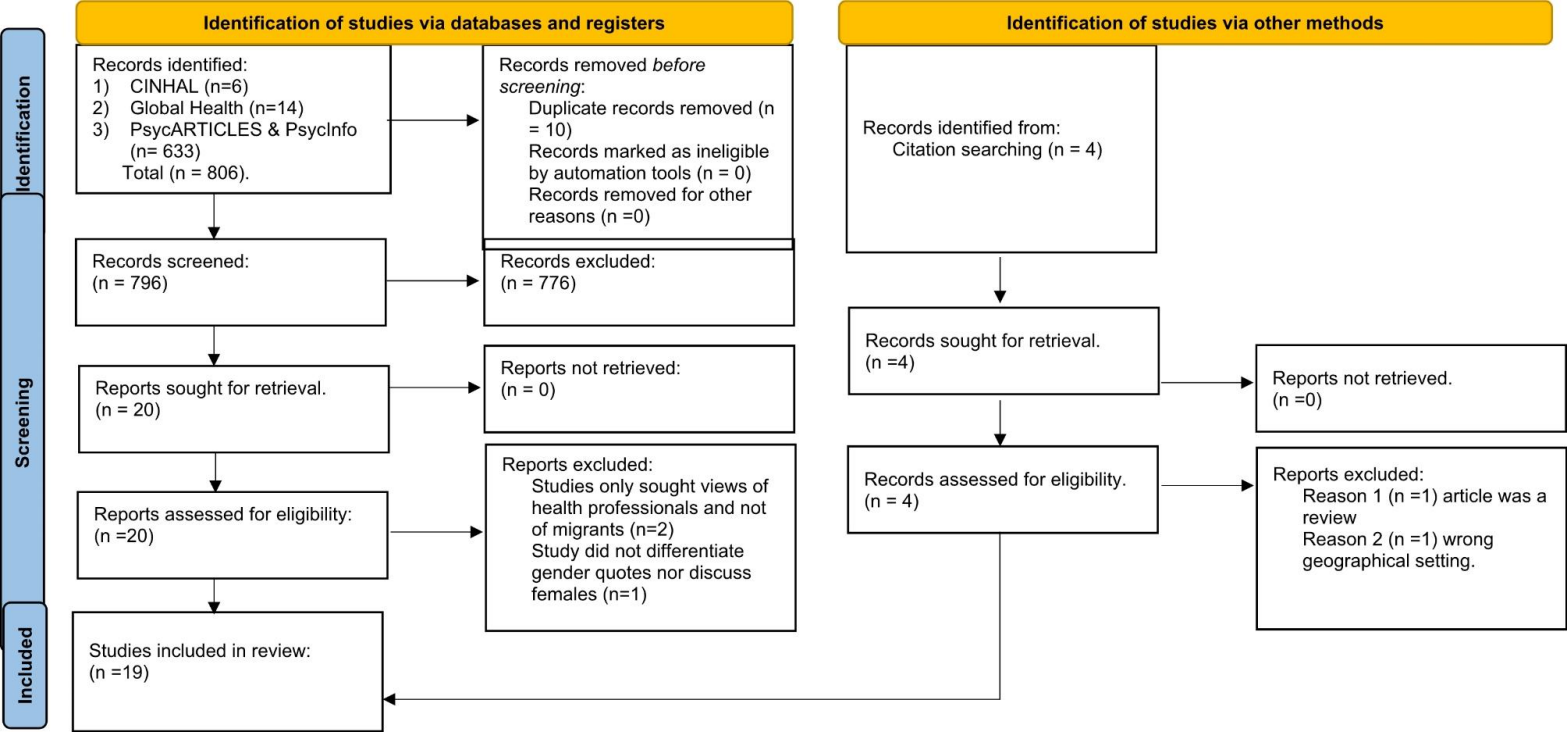
Prisma Chart *From*: Page MJ, McKenzie JE, Bossuyt PM, Boutron I, Hoffmann TC, Mulrow CD, et al. The PRISMA 2020 statement: an updated guideline for reporting systematic reviews. BMJ 2021;372:n71. doi: 10.1136/bmj.n71. For more information, visit: http://www.prisma-statement.org/

Of the 806 papers identified through database searches, 20 titles underwent independent full-text review by two reviewers (PN and AT), of which 17 were included in the review (Fig. 1). Many of these studies were excluded because they did not meet the inclusion criteria, such as not being written in English, not having a primary focus on mental health, or only including secondary care data. A small number of studies were also excluded because they were not relevant to the research question or because they were duplicates.

On further screening, two studies were excluded because they did not involve the collection of data from migrants. One study was further excluded from the final analysis because it did not differentiate data gathered according to gender. An updated search identified four further studies for consideration. Two of these studies were rejected because they did not meet the criteria to be included (being a review or being conducted in the wrong geographical setting). Disagreements between reviewers PN and AT about paper inclusion were resolved by discussion and, when needed, the involvement of other reviewers. As a result of these discussions, a further 2 studies were included in the final review. The full list of excluded studies and the process of identifying the studies for inclusion in this review are included in the PRISMA flow diagram (Fig. 1).

Key study characteristics were extracted and summarised (Table 2). Included studies were conducted in Austria, Finland, Germany, Italy, Norway, Sweden, Switzerland, The Netherlands, and the UK. One study [82] was conducted in several European countries including Slovenia, Croatia and Hungary and Italy. A further study included data from Syrian refugees in Egypt, Sweden, and Germany. This article was included in the review as its primary research countries were in Europe [83]. Eight papers were qualitative [82, 84–90], six papers were quantitative [91–96], with the remaining five papers utilising mixed method approaches [16, 83, 96–98].

**Table 2:** Study Characteristics

Author, year, country	Study design and setting Study setting	Participants Characteristics	Migrant Status Definition	Barriers and facilitators	Key Findings	% of females in the study
Markova et al. (2020) Norway [93]	**Design**: Quantitative **Setting**: Direct contact through digital means was used, including social media platforms.	**Participants**: Native comparison Norwegian students (*n* = 250) Russia (*n* = 151), Poland (*n* = 109), Pakistán (*n* = 117), Somalia (*n* = 100),	non-provided	**Facilitators:** Religious leaders Traditional and informal sources of support (friends etc.) as gatekeepers to support. **Internet forums**	Traditional help sources for MH were endorsed more by immigrants from Pakistan and Somalia than any other immigrant group in the study of native people from Norway.	G1*=69% G2 = 87% G3 = 77% G4 = 69% G5 = 44%
Linney et al. (2020) UK [86]	**Design**: Qualitative focus group **Setting**: Community-driven, co-produced with the Somali community in Bristol to address rising suicides within the Somali community in Bristol	**Participants**: Focus groups were held with separate groups for men and women N = 23 m (n = 12) f (n = 11)	non-provided	**Barriers**: Stigma Language barriers, lack of continuity and long waiting times. Lack of knowledge of MH illnesses. **Facilitators**: education, training, and awareness Increased services and older Somalis to talk to	The community provided ideas for improvements in mental illness recognition and accessing culturally safe support services	47.8%
Kiselev et al. (2020) Switzerland [85]	**Design**: Qualitative **Setting**: The study was part of the STRENGTHS project ^*f*^, evaluating the adaptation, implementation and scaling up of Problem Management Plus (PM+)	**Participants**: (*n* = 5) Healthcare providers (*n* = 5) and stakeholders (*n* = 5)	Syrian Key Informants - refugees and asylum seekers who had arrived after the outbreak of the Syrian war	**Barriers**: Language, gatekeeper-associated problems, lack of resources, lack of awareness, fear of stigma and a mismatch between the local health system and perceived needs	Multiple structural and socio-cultural barriers, with socio-cultural barriers being perceived as more pronounced.	60%
Mölsä et al. (2019) Finland [99]	**Design**: Mixed Methods **Setting**: Somalis living in Helsinki with matching to Finnish pairs through the National Register.	**Participants**: 128 Somalis, f(*n* = 75), m(*n* = 53) 128 matched Finnish pairs, f(*n* = 75), m(*n* = 53) All participants between the ages of 50–80	non-provided	**Barriers**: language, health professionals’ ignorance and insensitivity. Lack of knowledge of services, the stigma of MH within Somali society. Structural inequalities – Somalis did not have access to private doctors. Facilitators: sheikhs and imams	The Somali group had significantly lower access to personal/family doctors at healthcare centres. They used more nursing services than Finnish patients. Preference for traditional care, most commonly religious healing, for MH problems by most Somalis.	58.5%
Grupp et al. (2019) Germany [96]	**Design**: Mixed Methods **Setting**: A survey using paper-and-pencil and online assessments. approached in their accommodation facilities.	**Participants**: *n* = 119 asylum seekers from seven Sub-Saharan African countries, mainly Eritrea (*n* = 41), Somalia (*n* = 36), and Cameroon (*n* = 25). Each focus group had around 50% females.	Asylum seekers had to have flight experience and an origin in a Sub-Saharan African country.	**Barriers**: structural and cultural barriers to seeking medical and psychological treatment. Lack of knowledge of services **Facilitators**: Family and friends, religious leaders, preference for G.Ps.	Asylum seekers showed a high intention to seek religious, medical, and psychological treatment for symptoms of PTSD. Higher preference to seek help from religious authorities and GP.	**±** 30%
Kohlenberger et al. (2019) Austria [100]	**Design**: Quantitative **Setting**: Captured from a nationally representative survey of the population of Austria, Austrian Health Interview Survey (ATHIS) and Refugee Health and Integration Survey (ReHIS).	**Participants**: 515 persons Characteristics: 18–61 years Syrians (54%) Iraqis (16%) Afghans (23%) Other citizenship (7%) Gender: F(*n* = 73), M (*n* = 447)	non-provided	**Barriers**: conflicting schedules, long waiting lists, lack of knowledge, language problems **Facilitators**: High usage of day-care services	Refugees used hospitals and day-care services more than the average Austrian but less specialised services afterwards. Women reported more use of services than men and more unmet needs than men.	14%
Straiton et al. (2019) Norway [36]	**Design**: Quantitative **Setting**: National register-based cohort study utilising dynamic population - women living in Norway between 2009–2013 and diagnosed with at least one mood disorder were included.	**Participants**: Age: 16–67 years 1,834,822 women	Migrant - Born outside of Norway with two non-Norwegian born parents. Descendant - born in Norway, with two non-Norwegian born parents. The majority - all other women, including Norwegian born with at least one Norwegian parent and foreign-born with at least one Norwegian parent)	**Barriers**: stigma, language differences, the Western conceptualisation of MH disorders, consultation fees. **Facilitators**: length of stay likely to lessen barriers to access.	Migrant and descendant women were less likely to use outpatient MH services. Migrant women had fewer follow up consultations for their MH compared to the descendant and majority of Norwegian women.	100%
Carruthers & Pippa (2019) UK [101]	**Design**: Quantitative **Setting**: Data from two G.P. practices in South London.	**Participants**: (n = 35) Male (n = 20) Female (n = 15). Mean age = 35	Identified asylum seekers and refugees	**Barriers**: language issues and lack of interpreters, stigma, immigration concerns and information sharing.	High frequencies in psychiatric problems in refugees and asylum seekers but lesser referrals and use of secondary care compared to the UK average.	42.8%
Burchert et al. (2019) Germany, Sweden, and Egypt [83]	**Design**: Mixed Methods **Setting**: Step-by-Step (SBS) designed by the WHO ^*c*^ to help Syrian refugees access health systems in host countries.	**Participants**: n = 36 An equal number of men and women were interviewed in their host country. Mean age = 33.8 years (*SD* = 10.9)	Non-provided	**Barriers**: unacceptance of MH problems low technical literacy Lack of trust in apps Limited language skills High cost of smartphones and mobile data packages **Facilitators**: training and tutorials	Findings indicate the potential of e-health interventions in supporting the MH of refugees.	50%
Van Loenen et al. (2018) 7 EU Countries [82]	**Design**: Qualitative **Setting**: Fieldwork conducted in refugee reception centres in Greece, Slovenia, Croatia, Hungary, the Netherlands, Italy, and Austria	**Participants**: - 98 refugees: male (n *=* 65), female (*n* = 33) and 25 - 25 Healthcare workers: male (*n* = 9) and female (*n* = 16)	Refugees and other migrants without permanent residence permits	**Barriers**: Lack of information, lack of trust, time pressure, stigma, complex health and administrative systems, lack of continuity of care, language differences, gender, and culturally specific access to health care. **Facilitators**: interpreters and culturally competent health providers.	Refugees wished for compassionate health care provision and formal interpreters. They also hoped for information on healthcare provision and health promotion.	33.6%
Fox & Hiam (2018) UK [89]	**Design**: Qualitative **Setting**: Case Studies	**Participants**: Three females, Mariam (28), originally from Eritrea, Josephine (37) who fled from Uganda and Deidre, from The Caribbean. Both are identified as refused asylum seekers.	Separate box of definitions for immigration status including: **Refugee**: Someone whose asylum application has been successful; the Government recognises they are unable to return to their country of origin owing to a well-founded fear of being persecuted for reasons provided for in the Refugee Convention 1951 or European Convention on Human Rights. **Refused asylum seeker** person whose asylum application has been unsuccessful. **Asylum seeker**: A person who has left their country of origin and applied for asylum in another country but whose application has not yet been concluded.	**Barriers**: Hostile environment policies and practices, Lack of proper information and knowledge on rights of asylum seekers and failed asylum seekers by bother providers (G.P.s and asylum or failed asylum seekers). **Facilitators**: Doctors of The World, Red Cross, and churches	Recent policy changes compromise the healthcare needs of refugees, asylum seekers and failed asylum seekers.	100%
Papadopoulos et al. (2004) UK [16]	**Design**: Mixed Methods **Setting**: Estimated 25,000–30,000 Ethiopian refugees in the UK at the time of the study. The study applied a multi-method participatory approach which included members of the Ethiopian community.	**Participants**: Ethiopians resident in the UK (*n* = 106)	Asylum seeker - a person who has applied to the IND* to be recognised as a refugee but who has not yet received a decision or is in the process of appealing against an initial rejection of his or her claim.	**Barriers**: language problems Poor understanding of primary healthcare support. Postmigratory stress	Culturally competent services should be provided to migrants as postmigratory stress can lead to poorer health outcomes.	52%
Pooremamali & Eklund (2017) Sweden [95]	**Design**: Quantitative **Setting**: Sweden has two types of day centres accessed by people with MH: meeting place-oriented and work-oriented centres.	**Participants**: (*n* = 125) Immigrant background (*n* = 56) Native Swedes (*n* = 69) Migrants living in Sweden 11–45 yrs. (M = 27, SD = 9). Country of origin (*n* = 29) Born in Sweden but considered migrant (*n* = 15)	Being born outside of Sweden and/or having at least one parent born in another country’’ ‘’Immigrant background’’	**Barriers**: low educational attainment, disempowerment, low self-esteem, dissatisfaction with everyday activities. Stigma and discrimination **Facilitators**: Integration due to length of stay.	Being of immigrant background and having an MH illness was a negative factor to empowerment.	59%
Morgan et al. (2017) UK [94]	**Design**: Quantitative **Setting**: The UK is host to an increasing refugee and migrant population, however, continues to put restrictions on them on employment, housing, benefits, and detention for some during the process.	**Participants**: (*n* = 97) Mean age 33.8 (SD = 8.4), range 18–59 years 57% refused asylum (*n* = 55) Countries of origin (*n* = 25) Female (*n* = 46), Male (*n* = 51)	non-provided	**Barriers**: Financial, housing, Unsecure immigration status, Isolation **Facilitators**: information on acculturation including English language classes, Perceptions of democracy and freedom.	Both sets of participants, asylum seekers and those who were refused asylum reported levels of anxiety, stress, depression, and PTSD. Those who were refused asylum scored higher on depression and anxiety.	47%
Ali et al. (2016) UK [84]	**Design**: Qualitative **Setting**: Lower referral rates to CAHMS ^*b*^ for children from ethnic minority backgrounds. Pakistanis make up the largest ethnic minority in Peterborough.	**Participant ages**: 11-19yrs. Four focus groups (FG). FG1 - boys (*n* = 10) FG2 – girls (*n* = 7) FG3 boys (*n* = 7) FG4 – girls (*n* = 9)	Participants held or were descendants of Pakistani passport holders. Parents were in transnational marriages.	**Barriers**: Lack of information on accessing support, stigma **Facilitators**: Religious leaders, mentoring schemes with older students and information from the internet	Participants had poor awareness of MH services and treatment options. Culturally appropriate awareness of MH and support that was gender-specific were suggested.	48.4%
Loewenthal et al. (2012) UK [87]	**Design**: Qualitative **Setting**: Bengali, Urdu, Tamil, and Somali speaking communities recruited through their community associations	**Participants**: **Bengali**: 1st focus group f (n = 8). 2nd focus group f (*n* = 4) m (*n* = 2) **Urdu**: 1st focus group f (*n* = 15), 2nd group, m (*n* = 6) **Tamil**:1st group m(*n* = 10) 2nd group f(*n* = 8) **Somali**: 1st group f(n = 14) 2nd group m(*n* = 10) Validation interviews: **Bengali** f (*n* = 4) m (*n* = 2), **Urdu** f(*n* = 3), m (*n* = 3), Tamil m(*n* = 4), f(*n* = 2), **Somali** m(*n* = 3), f(*n* = 3)	non-provided	**Barriers**: Understanding of MH issues and availability of MH services Cultural barriers Stigma Disclosure of MH problems **Facilitators**: community-based interventions. Awareness-raising forums. Religious leaders	Participants did not fully understand common conceptualisations about MH issues, nor did they know how to seek mental health support.	59.2%
Tabassum et al. (2009) UK [102]	**Design**: Qualitative **Setting**: The study was conducted in Darnall, Sheffield, with high unemployment and deprivation with few white residents. Interviews were held in participants homes. Females were interviewed with the whole family present due to cultural considerations.	**Participants**: Males (*n* = 22) 1st Generation females (n = 29) 2nd Generation females (*n* = 23) Four individuals did not participate due to a lack of conceptual knowledge of mental health (m = 1, 1st gen f = 1, 2nd gen f = 2)	First-generation women born and grew up in Pakistan. Second-generation women born and grew up in the UK.	**Barriers**: lack of proficiency in English Stigma Isolation due to cultural proscription Racism Reluctance to involve others in support. **Facilitators**: faith healers, G.P. and Family support	The western conceptualisation of MH may not be the same as Pakistani understanding. Stress at home was cited as the most likely cause of mental illness. G.P consultations were favoured for accessing support, particularly by males, though the emphasis was on physical health symptoms even though it may have been for mental health.	70.2%
Whittaker et al. (2005) UK [90]	**Design**: Qualitative. A cross-sectional study of participant individual and group interviews **Setting**: Participants were recruited from a Somali community centre	**Participants**: Five females. Females born in Somalia and who had been resident in the UK since they were children or adolescents. Additionally, participant born in the UK was included and analysis and discussion were provided separately as part of enriching the study.	Female refugees born in North Somalia. To the participant born in the UK: “not a refugee but was born in the UK to a refugee family”.	**Barriers**: Religion, the intersection between culture and religion, stigma **Facilitators**: Resilience, religion family and community	Intersections of religion and culture may hinder access to support. The complexities of approaching services due to fear of disclosures, stereotyping and individual beliefs are clinical implications in providing service options.	100%
Knipscheer & Kleber (2001) The Netherlands [97]	**Design**: Mixed Methods **Setting**: Recruitment through two summer festivals in Amsterdam and The Hague. Additional data was gathered through outpatient MH* services.	**Participants**: Study 1: Surinamese citizens in the general population (*n* = 292) m (*n* = 163), f (*n* = 129) Study 2: Surinamese (Hindustan Surinamese Dutch, Creole Surinamese Dutch, mixed Chinese, and Javanese background) and inclusion of 89 indigenous Dutch for comparisons. F (*n* = 145), M (*n* = 40)	People who have recently migrated from Surinam to the Netherlands.	**Facilitators**: Familiarity with community MH centres Friends and family **Barriers**: low education Prejudice and misconceptions about CMHC. Lack of support information.	Length of residence is an important predictor of both behaviour and attitudes, with the more recently migrated most in need of education on the utility of Dutch MH services.	S1 = 44% S2 = 78%

Terms used in the table

MH* = Mental Health; CAHMS ^*b*^ = Child and Adolescent MH Services; WHO ^*c*^ = World Health Organisation; *IND = Immigration and Nationality Directorate; GP ^*d*^ = General Practitioner; PTSD ^*e*^ = Post Traumatic Stress Disorder; The STRENGTHS project ^*f*^ = Scaling up psychological interventions with Syrian Refugees; IAPT ^*g*^ = Improving Access to Psychological Therapies; BAME ^*h*^ = Black, Asian, and Minority Ethnic; CBDC ^*i*^ = Community-based day centres; G1*, G2* = Group 1, Group 2…; S1*, S2* = Study 1, Study 2…

The studies with the highest proportion of female migrants were 100% ([36, 89, 90], and the study with the lowest proportion of female migrants was 14% [91]. The study with the largest number of participants was the study by Straiton et al. [36], with 1,834,822 women. The study with the smallest number of participants was by Fox and Haim [89], with 3 participants all of whom were female. Qualitative methods were used in most of the studies, with 8 studies [82, 84–87, 89, 90, 99] using this design. Quantitative methods were used in 6 studies [36, 91–94, 95], and mixed methods were used in 5 studies [16, 83, 96, 97, 98].

Community-based settings were the most common setting for data collection, with 11 studies conducted in this setting [16, 83–87, 90, 92, 96, 97, 99]. Primary care settings were used in 5 studies [36, 82, 89, 92, 95], and a mixture of primary care and community settings was used in 3 studies [93, 94, 98]. However, it is important to note that the definition of primary care is subjective, and some studies may have used either or both settings of community and primary care.

### Mixed methods appraisal tool (MMAT)

There were no significant differences in the studies independently assessed using the Mixed Methods Appraisal Tool (MMAT) by the two reviewers involved in the appraisal process (AT and PN). As per the study design, no papers were excluded due to study quality. All the papers were assessed as having good study quality. The results of the appraisal are presented below (Table 3).

**Table 3:** MMAT Scoring

	Qualitative				
	1.1. Is the qualitative approach appropriate to answer the research question?	1.2. Are the qualitative data collection methods adequate to address the research question?	1.3. Are the findings adequately derived from the data?	1.4. Is the interpretation of results sufficiently substantiated by data?	1.5. Is there coherence between qualitative data sources, collection, analysis & interpretation?
Ali et al. (2016)	Y	Y	Y	Y	Y
Kiselev et al. (2020)	Y	Y	Y	Y	Y
Linney et al. (2020)	Y	Y	Y	Y	Y
Loewenthal et al. (2012)	Y	Y	Y	Y	Y
Tabassum et al. (2009)	Y	Y	Y	Y	Y
Van Loenen et al. (2018)	Y	Y	Y	Y	Y
	Quantitative				
	4.1. Is the sampling strategy relevant to address the research question?	4.2. Is the sample representative of the target population?	4.3. Are the measurements appropriate?	4.4. Is the risk of nonresponse bias low?	4.5. Is the statistical analysis appropriate to answer the research question?
Carruthers & Pippa (2019)	Y	Y	Y	Y	Y
Kohlenberger et al. (2019)	Y	Y	Y	Y	Y
Markova et al. (2020)	Y	Y	Y	N	Y
Morgan et al. (2017)	Y	Y	Y	U	Y
Pooremamali & Eklund (2017)	Y	Y	Y	U	Y
Straiton et al. (2019)	Y	Y	Y	Y	Y
	Mixed Methods				
	5.1. Is there an adequate rationale for using a mixed-methods design to address the research question?	5.2. Are the different components of the study effectively integrated to answer the research question?	5.3. Are the outputs of the integration of qualitative and quantitative components adequately interpreted?	5.4. Are divergences and inconsistencies between quantitative and qualitative results adequately addressed?	5.5. Do the different components of the study adhere to the quality criteria of each tradition of the methods involved?
Burchet et al. (2019)	Y	Y	U	U	Y
Grupp et al. (2019)	Y	Y	Y	U	Y
Knipscheer and Kleber (2001)	Y	Y	Y	U	Y
Mölsä et al. (2019)	Y	Y	Y	U	Y
Papadopoulos et al. (2004)	Y	Y	Y	U	Y

Key: Y = yes, N = no, U = Unsure/Undecided/Unclear

### Advancing research on female migrants through lived experience

The female migrant coproduction group that contributed to the review discussed the barriers and facilitators to mental health care support. These concepts informed the research question and were the basis for the final themes. See Supplementary File 2 for the initial framework for analysis.

We aimed to assess the methodological approaches of including lived experience perspectives in migrant research. We specifically examined whether studies included lived experience perspectives in the study team or data collection methods. We only analysed the methods sections of studies to identify mentions of lived experience during data collection and excluded any mentions in the discussion sections. This allowed us to focus on the methodological approach of including lived experience in migrant research.

Several studies explicitly described the recruitment of co-researchers with lived migrant experiences in their methods Sects. [16, 83, 85–87, 90, 92, 96, 97, 98, 100]. For example, Papadopoulos et al. [16] recruited and trained eight Ethiopian research assistants who conducted all interviews in Amharic. Similarly, Burchert et al. [83] collected data using trained Arabic native speakers. Linney et al. [86] and Mantovani et al. [100] utilised community partners and trained community well-being champions, respectively.

## Theme summary and interpretation of results

The themes which were identified regarding barriers to accessing mental health support by female migrants in primary and community care were: lack of access to appropriate information, cultural barriers, stigma, and structural, and gender-specific barriers. Gender-appropriate/sensitive services were seen as enhancing the likelihood of access to support. GP services were seen as facilitators to access and support, as many female migrants had expressed a willingness to use the services. Further potential facilitators were identified including culturally appropriate services, gender-specific support, and religious leaders. The themes are summarised in Table 4.

**Table 4:** Key Themes and Sub-themes

Barrier Themes	Barriers Sub-themes
**Access to information**	• Individuals do not know where to obtain information. • Services not providing readily accessible information. • Services not aware of up-to-date information and guidance
**Cultural and Spiritual barriers**	• Religious and cultural practices • Religious and cultural beliefs • Intersection of religion and culture
**Stigma**	• Self-stigma of MH. • The societal stigma of MH, including that of family members with MH ill-health. • Institutional stigma, including negative beliefs of reasons why migrants access MH services
**Structural barriers**	• Service delivery does not consider gender. • Service delivery not supporting certain types of migrants. • Lack of interpretation services • ‘Hostile environment’ policies and practices
**Gender-specific issues**	• Poor quality of research papers informing service and policy. • Poor understanding of differences between gender and cultural needs when delivering MH support. • Prioritisation of male health needs • Prioritisation of male voices in research • Intersectionality
**Facilitator Themes**	**Facilitators Sub-themes**
**Religiosity, Community, and religious leaders**	• The willingness of migrant females to discuss mental and spiritual health. • Spiritual leader awareness of mental health conditions • Supportive friends and family
**Gender-sensitive support**	• Availability of culturally and gender-sensitive mental health support. • Peer support from other migrant females
**Education settings as facilitators**	• Subjects such as Psychology increased awareness of MH • Social aspects of educational environments increased chances to access support compared to isolated settings.
**Resilience and adaptability**	• Resilience as a factor in the increased likelihood of seeking support. • Adaptability and acculturation to a new environment.

## Barriers to seeking and accessing mental health support

### Accessing support information

Information access was highlighted as an issue by participants in two studies [85, 97]. A study conducted in the UK found limited information on the identification of mental health disorders and seeking community support amongst young school-aged Pakistani females: “*I don’t think they tell us like early enough, you know when you’ve found out then they tell you. I think there should be something where you find out before a little*.” (FG, Young female) [85]. This apparent lack of information was not confined to young school-aged females, as one 34-year-old female from Eritrea expressed frustration at not knowing whom to talk to about her mental well-being due to a lack of awareness around which services are appropriate to access for mental health support: “*Who is my contact person regarding this inner anxiety?*’’ [96]. Three further studies included observations by researchers on information availability and access as a barrier to support for mental health for female migrants [83, 89, 91, 99]. For older migrant women, the barrier to accessing information for their mental health using technology was two-fold: firstly, an inability to use modern technology such as a smartphone to search for information, and secondly, an over-reliance on sons or grandsons to access information through technology which meant the loss of privacy and independence [83]. Similarly, a study affirmed this notion by stating that most first-generation women had limited educational backgrounds, such as the minimum ability to read the Quran which they could not do, thus access to information that required reading was considered a barrier [99].

### Religious and cultural intersectional barriers

One study highlighted the dilemma young female migrants faced when engaging with male spiritual leaders within their communities in their host countries. On one hand, they could seek support for their spiritual needs, however, when it came to their mental health, gender was seen as a barrier as ‘‘*girls cannot talk to a man*’’ (FG2, Girl 5) [84]. The belief in spiritual manifestations to explain behaviour was seen as re-enforcing stigma and an overreliance on religious and societal explanations for poor mental health, for example, *“Those of us from a Black background…if anybody tells you that you have a mental health issue you are ready to fight them for saying that. I mean…because we relate mental health to insanity, a total level of insanity’’* (Female, African). Some of the related treatment options included prayer and whipping to drive off the evil spirits [84, 99, 100]. Younger migrants who traditionally rely on their parents for support had trouble adjusting to being independent in their host countries [16].

One study mentioned the impact of “*power relationships between men and women in Muslim culture*, *where men are generally dominant in the relationship and women are required to be more subservient”*. The study found that women were less likely to see a GP than men because they were often not “allowed” to do so by their male partners or family members. This reflects the traditional gender roles that are still prevalent in many cultures. The women were, therefore, less likely to seek support from and disclose their mental health issues to male GPS due to the prevailing gender power imbalances [99].

### Stigma as a barrier to support

Societal stigma was prominent in three studies [82, 84, 100]. Self-stigma and perceived stigma of mental health stopped young females from seeking support from family or services, “my friend…never tells her mom anything [about her mental health problems] and she always bottles it up and she just…thinks of her mum getting upset” (FG2, Girl 1) [84]. The stigma attached to being a migrant within health settings was highlighted, “the doctor, for example, is suspicious and thinks, all the asylum seekers are taking advantage of Switzerland and on top of that they fake being psychologically distressed.” (Stakeholder, Switzerland) [85]. Although younger female migrants are more likely to be socially active and have greater access to mental health support, they are also more likely to experience racism. This can be a cause of mental health problems, and it can also make it more difficult for them to access the support they need [16, 84, 99].

### Structural and service barriers

Service Delivery. Female migrants reported more unmet healthcare needs compared to their male counterparts [89, 91]. There was reluctance to access services due to suspicion of asking for help from strangers and when they did access services, female migrants reported that they often were not sensitive to their needs, specifically mental health services, and statutory services that the female migrants felt had the power to detain or deport individuals [16, 89]. Similarly, one study reported that female migrants are not likely to utilise some primary care services compared to their male counterparts in an area of the UK as most of the GPs were male [99]. Regarding the heterogeneity of definitions as to what constitutes a migrant, mental health support services too were considered inaccessible to certain types of migrants, for example, failed asylum seekers [89, 93]. Josephine, a failed asylum seeker, originally from Uganda, was pregnant and still could not register with a GP in the UK to access support for her physical and mental health, “Every time they would chase me away, they told me that as my visa was still valid, I wasn’t entitled. They told me I would have to pay something like £300” (Josephine, Uganda) [89].

Language barriers were identified in accessing psychological and physical support for female migrants both in transit to the host nation and upon arrival in the destination nation. As one female participant from Ghana who travelled to Italy stated, “*The doctor did not speak English, did not understand, then at some point spoke in Italian….*” (Female, 23, Ghana) [82]. A female Ethiopian migrant alluded to the relationship between unmet physical health needs and the impact they had on her anxiety, as she stated that it was only when her kidneys dropped to functioning below 5% that the GP who had never asked for a translator finally arranged for one [16]. One female mentioned, “*You don’t know how to approach that person who is not in your language speaking”* (Female, FG1, P1) [86].

### Gender-specific barriers

Several studies highlighted how traditional gender roles may play a role in accessing support for mental health needs. For example, Kiselev and colleagues (2020) mentioned a lack of childcare opportunities for female migrants may act as a barrier to support as it is always assumed the burden of responsibility for looking after children fall upon women. Additionally, they identified the burden female migrants experience in keeping their mental health concerns within their family rather than talking to health professionals “*One always says you can talk to your mother and with your friends. This is the way it is in Syria”.* The same burden is then placed on migrant mothers not accessing support for their mental health, “*maybe fathers are always outside having kind of fun thing or at least chatting to another person, so that is why it is not that many big issues, but when it comes to the mothers the problems are bigger larger scale”* [86]. One study reported how males did not want their female members of the family’s voices to be listened to by anyone else outside the family [99]. There was further reluctance for some males to let females access support for their mental health due to fear of the domestic burden at home being raised in the absence of the female [99]. Young Pakistani females felt they would not be able to discuss issues in a family therapy setting, *“you wouldn’t want to say anything…rather have one-to-one’’* (FG2, Girl 1). Older females tended to be socially isolated which in turn reduced access to support [83, 86, 99]. Female migrants who were pregnant, and had previously experienced gender-based violence, including forced marriages encountered maternal health issues which impacted their mental health [89].

## Facilitators to seeking and accessing mental health support

Although few facilitators for accessing support were identified, the role of charities was praised. One study participant stated that “*Nowadays there are so many charities that are helping*” (Female, Somalian, UK) [86]. In cases of female migrants whose immigration status meant that they were limited in support from traditional health care, doctors could also refer them to charities that were sympathetic and could meet migrant mental health needs [89]. Additionally, a greater focus on awareness was seen as an essential part of enabling migrants to access support, as one female migrant asserted, “*Awareness is the first and the most, educating the community, understand that this is an illness.*” [86].

### GPs

Several studies [16, 86, 87, 92, 97] considered GPs as accessible for female migrants when dealing with their mental health needs, “*Whatever problem we have in mind, the first person to contact will always be the GP.*” (Female, Somalian, UK) [86]. Though traditional GP surgeries may have refused to provide support for some undocumented or refused asylum seekers, doctor-led organisations such as Doctors of the World filled the role of providing them with easier access to support [89]. Moreover, one study highlighted the role of community mental health support for female migrant mental health needs, with greater satisfaction reported than their male counterparts [97].

### Technology

For younger female migrants, access to appropriate technology such as smartphones and the internet was seen as positive support, *“I know that there is ChildLine where you just speak …”* (FG, Girl 5). Culturally sensitive healthcare support, especially when delivered by female migrants, was identified as a facilitator to access and support [84, 86, 97]. Equally as important was the provision of readily available interpretation services within some GP surgeries, “*having an interpreter stand by is always good…where you have access…9 to 5 is always a big point. Yeah, well done to the [GP surgery]”* (Female, FG1, P4) [86].

### Religiosity, community, and religious leaders

Having access to spiritual leaders and spiritual support from friends and family was a demonstration of the intersection of religion and culture in alleviating symptoms of distress [16, 89, 90, 96]. This was the case, particularly for older migrants who looked up to imams and sheikhs to alleviate their mental health distress [98]. Relatives were seen as possible facilitators to support, as Zeta explains, *“And lots of relatives…so I won’t feel lonely…they visit us quite often”* [90].

### Educational settings

The role of education in providing knowledge was considered an enabler for awareness of treatment and support options such as Cognitive Behavioural Therapy (CBT) for young female migrants, with subjects such as Psychology cultivating awareness in this demographic [84]. Young female migrants were able to socialise with their friends and could speak to them openly about mental health, unlike most of their older female migrant colleagues.

### Resilience and adaptability

Three studies highlighted feminine resilience and adaptability [16, 89, 90]. Females were more likely to successfully adapt and be more socially active which could lessen their chances of developing mental health conditions due to loneliness and isolation. Increased social integration ensured that they learnt English quicker than their male counterparts, and thus were more likely to start or continue in education and be employed. Where challenges were faced, female migrants persevered, as illustrated by the stories of Josephine and Miriam [89] and Aisha, who states, “*The way I see Somali woman is, is that they are really strong… Most of them are single mothers, and the way they cope is unbelievable, it’s unbelievable. I mean, they try their best, yeah? And most of them don’t have any families around at all, it’s only them.”* (Aisha, Group 2) [90].

### Feminist appraisal of the included papers

Study characteristics and a summary of quality using the Feminist Appraisal Tool are presented (Table 5).

**Table 5:** Feminist Appraisal of Studies

	Feminist appraisal		
Author and Year	Study Conceptual Underpinnings*	Gendered Context in Discussion	Quality of feminist analysis
Ali et al. (2016)	N/A*	Researchers state gender mixing is not socially prescribed in Pakistani culture hence the reason for holding separate focus groups between boys and girls.	Cursory*
Burchet et al. (2019)	N/A	Previous research has shown that access to expensive communication devices tends to vary along age and gender lines. Older women often relied on their sons or grandsons when it came to the use of communication technologies (24).	Cursory
Carruthers & Pippa (2019)	N/A	N/A	Cursory
Fox & Hiam (2018)	N/A	N/A	Cursory
Grupp et al. (2019)	N/A	Less frequently cultural barriers in accessing healthcare were mentioned predominantly by female participants preferring female doctors and if possible	Cursory
Kiselev et al. (2020)	N/A	Other barriers such as lack of childcare opportunities for women and transport costs were mentioned once each.	Cursory
Knipscheer and Kleber (2001)	Adjusted for age and gender,	Women made relatively more use of the CMHC than men – they reported more MH problems and had greater satisfaction with CMHC services	Cursory
Kohlenberger et al. (2019)	N/A	Unmet health needs and barriers to health access are relevant concerns for recently arrived refugees. Female refugees below 40 years of age report worse health than Austrian women. In	Thorough*
Linney et al. (2020)	N/A	N/A	Cursory
Loewenthal et al. (2012)	N/A	Due to cultural considerations, the four researchers, all of whom were themselves born outside of the UK and, in terms of their languages and cultural backgrounds, members of the respective communities relevant to this study, conducted the focus groups and respondent validation	Moderate*
Markova et al. (2020)	N/A	N/A	Cursory
Mölsä et al. (2019)	Almost 48% of Somali language speakers were female in Finland.	Somali women used less preventive healthcare as compared to other female migrants.	Cursory
Morgan et al. (2017)	N/A	N/A	Cursory
Papadopoulos et al. (2004)	N/A	N/A	Cursory
Pooremamali and Eklund (2017)	N/A	N/A	Cursory
Straiton et al. (2019)	Comparing migrant and descendant women’s use of OPMH services with the majority women using national-level registry data.	Overall, our results suggest that migrant and descendant women use OPMH services to a lesser extent than most women. Descendant women are less likely to use OPMH services, while migrant women are both less likely to use OPMH services and have fewer follow-up consultations for common MH disorders.	Moderate
Tabassum et al. (2009)	A secondary aim of exploring the needs of women, for MH services.	N/A	Moderate
Van Loenen et al. (2018)	N/A	Less frequently cultural barriers in accessing healthcare were mentioned predominantly by female participants preferring female doctors and if possible, from the same geographical/cultural background.	Cursory
Whittaker et al. (2005)	Exploring individual and collective understandings of psychological well-being among young Somali asylum-seeker or refugee women.	The findings of the research highlight the tensions for the women participants when religious interpretations were used to constrain gender roles	Moderate

Conceptual underpinnings* = definitions of gender and epistemologies study authors are influenced by the methodology

N/A* = Not clear or not clearly stated. This has been used throughout this table to signify information lacking enough to be analysed within the scope of this review

Cursory = satisfying one category of the tool

Moderate = satisfying 2–3 categories of the tool

Thorough = Consideration of gender and power as measured against the tool’s framework

Only three of the included studies [36, 90, 91] implied a conceptual underpinning of the study concerning gender as a study outcome. Overall, there was little consideration of possible power imbalances between the researcher and participant groups of different genders. Where studies sought specific views from females, male presence in the form of a family member was justified as being normal within non-Western cultures [99]. Where the views of both male and female participants were included in the data analysis, several studies chose to highlight male issues to the detriment of female issues through their use of supporting quotes. For example, one study with close to equal numbers of male and female participants included 22 quotes from male participants and only one from a female participant [96]. The researchers acknowledged this as a weakness of their study and recommended that future research should place more emphasis on female voices. Further recommendations for improving access, support and producing higher quality research concerning females are suggested by some papers [36]. One study included gender and sex as a category in the analysis [94].

Overall, only one paper [91] had a ‘thorough’ feminist analysis of the four categories of the framework. Four papers scored moderately [36, 87, 90, 99] meaning that they had satisfied 2–3 of the above categories, whilst the rest of the papers had a score of ‘cursory’ meaning that they had satisfied at least one of the above categories. Qualitative papers were stronger in quality compared to mixed and quantitative methods.

Cultural sensitivities being put at the forefront of gender issues ran the risk of biased reporting; one study [96] reported the risk of response bias and social desirability within a participant focus group which was male-dominated and facilitated by a White researcher of the majority population. There are no considerations about female participants being included in a group of mostly males and the effect this group composition would have on females’ motivation to speak about their intentions and beliefs around treatments for PTSD. The inclusion of the sex and gender of the researchers may have implications on the interpretation of the studies when accounting for the sex and gender of the researcher and their interactions with the participants.

Some studies included in the review, for example, reported gender differences and similarities in perceptions and experiences in focus groups and interviews [16, 84, 86, 87, 92, 98, 99]. However, the study participants included in most of the studies had one defining characteristic, that of low socioeconomic status, regardless of migrant background. This runs the risk of not identifying the needs of the fewer female migrants who are not of low socioeconomic status. The resilience of female migrants is often not acknowledged in studies, however, for a population deemed ‘’at-risk’’ and facing insurmountable challenges in a traditionally hostile environment for migrant populations, resilience and adaptability were key in overcoming challenges with integration. For example, in one study [16], Ethiopian women adapted better to life in the UK than their male counterparts due to what they termed as feeling liberated from positions of subjugation, thus becoming more active in public life. This can be contrasted with older Pakistani women, who felt socially isolated [99].

## Discussion

The findings from this review suggest that female migrants face gender-specific barriers to help-seeking and accessing mental health support during migration, post-migration, and acculturation. In the review by Gebremeskel and colleagues [101], which looked at the barriers refugee women face in accessing mental health services in high-income countries, stigma, language, lack of culturally appropriate support and gender roles were found to be barriers to access. Their results share some similarities with the results of the current review. However, the review by Gebremeskel centred on refugee women, whereas the current review includes other forms of migrancy. Our findings also highlight the poor quality of migrant research where issues relating to female needs are not fully addressed or are simply left out entirely. The lack of framing of gender within included studies impacted the way gender was analysed. For example, the studies did not address the challenges of defining sex and gender and what impact a lack of consideration for this would have when reporting different sex and gender needs. To the best of our knowledge, this systematic review is the first to analyse the quality of migrant studies using a feminisms lens to examine female migrant access to mental health services in Europe. The dual effect of GPs and religious leaders being both barriers and facilitators to access for female migrants poses serious concerns as well as opportunities for female migrants who may attempt to access both for their, mental, physical, or spiritual support needs. Little is known about the impact of both being facilitators and barriers.

The current review confirms, as previously reported, how inequalities in gender roles may act as a barrier in addition to other socio-cultural barriers and labels associated with being a migrant. This is consistent with a plethora of studies that have reported similar results [102–104]. The case studies of Mariam, Josephine and Deidre highlight the complexities of dealing with female migrant cases. For example, one individual can present with multiple experiences and labels, which all intersect. In Mariam’s case, a Black African female, widow, mother, a victim of rape, violence, smuggling, refused asylum seeker, homeless and with poor physical and mental health states [89].

The included studies implicate a lack of information awareness as a barrier to accessing support for mental health conditions by female migrants. Furthermore, stigma was identified as a barrier to accessing support. Some migrant mothers expressed a reluctance to discuss mental health concerns for fear of deportation or their children being taken away by social services, whilst some who were pregnant were turned away from primary care services in host countries with a policy of access for all to primary healthcare. These fears are echoed in similar reviews that identify this fear as a barrier to accessing support specifically among undocumented migrant mothers and expectant mothers [26, 27]. This highlights the differing needs and perceived rights of different migrant populations, and therefore, the weakness of blanket information awareness policies and strategies in increasing access to support which are not tailored to or appropriate for the needs of migrant women. The results demonstrate why the rigour of migrant research should be critically examined, as it has implications for service provision and information awareness for female migrants, regardless of migrant status. Country of origin may affect the perceptions of accessing and delivering services to female migrants. For example, the linguistic minority in Italy have found it difficult to access support due to not speaking Italian. Similar findings were reported in a review of refugee and asylum seeker health in Europe [47].

Language has long been considered a barrier to finding employment and accessing health support for migrants [28, 105–107]. Language difficulties and cultural proscriptions on socialising led to social isolation and a lack of integration within host communities for some older female migrants. Additionally, the distressing experiences of female asylum seekers being assisted by a male interpreter when accessing support for their health to circumnavigate this barrier have been reported previously in other reviews [28, 105]. Acculturation has been identified in a review by Lindert et al., as a factor for service utilisation for migrants [12]. Curiously, women with a low educational level who had a longer residential stay sought treatment more often, alluding to presenting with mental health needs later than other populations [97]. In the current review, female resilience and adaptability were shown as factors in overcoming religious, cultural, structural, and social barriers and therefore promoting post-traumatic growth. This encouraged some female migrants to seek support.

### Research implications

One paper [96] raises serious questions for policymakers regarding population management and health, particularly as the paper is heavily influenced by 22 quotes from male migrants and only one quote is attributed to a female migrant. The review raises questions about the impact and applicability that migrant research findings have on service design. Most migrant studies involve younger migrants, but questions are still left as to what happens to the female migrant population as it ages when considering access to support. The review has demonstrated that equal representation of female migrant participants in studies does not always equate to equal reporting of findings where gender is concerned. To the best of our knowledge, this is the first review to make that connection.

For the feminist appraisal, our findings are similar to results obtained in a feminist quality analysis which indicated the relative strength of qualitative studies compared to quantitative studies when scoring the feminist appraisal tool [79]. Exploring the studies using a feminist lens has enabled the review to include personal facilitators of resilience and coping which are often missed when exploring barriers and facilitators to healthcare. Additionally, this review calls into question the prioritisation of migrant status over gender and sex and why intersectionality is seldom considered in migrant studies.

### Implications for public health and primary care policy and practice

Evidence gathered to support policy on migrant mental health should consider the heterogeneity of migrant populations. Migration studies should include a fully operationalisable definition of migrancy and the impact of the definition on studies. More people need to know about the mental health services that are available to female migrants. Referral processes for mental health support need to be clear on the needs identified concerning gender-specific needs. Within the support organisations, staff must be trained to consider the gender-specific needs of female migrants, including the impact that social and physical health inequalities have on their mental health. Commissioners of services should require potential services supporting migrants to consider gender-sensitive service delivery. There are gaps in research on failed female asylum seekers in accessing services. Given the higher risks of developing or exacerbating poor mental health, health promotion should aim to increase access to support. Failed asylum-seeking females were rarely mentioned in studies, as was the difference in access for asylum-seeking females and other forms of migrants. Studies have mostly focused on female migrant populations with low-socioeconomic status, as these individuals tend to be unemployed or in low-paying jobs. However, efforts must be made to better understand the mental health needs of the minority of migrant females that may not fall into this category, specifically on their resilience in often high-stress environments and male-dominated roles. The high prevalence of perinatal depression, PTSD, and anxiety in the general female migrant population warrant the development of specialised mental health services tailored to the specific needs of this group of migrants. Female migrant research, policy, and commissioning must address the individual, social, and structural factors that affect access to and support specific to female migrant mental health. The review has highlighted some of these factors which include trauma, social isolation, and stigma, which are all rooted in intersecting systems of oppression that this specific group of women and girls face. Promoting feminist participatory action research may help overcome some of the obstacles which traditional research has encountered in engaging with migrant populations.

### Strengths and limitations

To the best of our knowledge, this is the first review to employ a feminist lens to critique the quality of migrant studies by highlighting shortcomings that may be used to inform immigration policies and public opinion in Europe. A strength of the current review is the geographical breadth of the included studies, which has highlighted a need for coordinated and concerted efforts in planning and policymaking to lessen barriers to access to support for mental health by female migrants. Specific migrant laws are diverse across countries; therefore, this could be viewed as a weakness of the review as it does not consider country-specific migratory policies. Further, it is acknowledged that no paper was rejected for review based on quality. An additional acknowledged weakness of the review is the inclusion of research that focused primarily on male migrants whilst overlooking female issues, with a sparsity of quotes representing female migrants. Though it is a strength in critiquing a male narrative, it is open to criticism for not fully raising feminist issues to access mental health support in primary care. The lack of a standard definition of “migrant” across the studies could be a limitation of the review. The definition of “migrant” may not have been consistent across the included papers and possibly a mismatch with the definition in the review. This could lead to inconsistencies in the data and make it difficult to draw firm conclusions. Naming specific services and therapies, (e.g., CBT and psychotherapy) may have had the effect of narrowing the search, likely causing relevant articles to be missed. Further weaknesses of the review include the exclusion of studies not written in English and the exclusion of grey literature due to a large amount of policy and NGO data on newcomer populations in Europe. This raises an issue about inclusivity in research when searches are strictly narrowed down to studies written in English and peer-reviewed articles.

This review is limited in its focus on general practitioners (GPs) and other medical agencies, rather than the broader range of mental health primary care providers, which also includes pharmacists. Future research should examine the role of a wider range of primary care providers as both barriers and facilitators to mental health care.

## Conclusions and recommendations

Compared to the general population and male counterparts, female migrants face a greater number of barriers to accessing support for their mental health needs in primary care. Future research should consider gender and its impact on design, recruitment, interviewing, interpretation of results and intervention formulations in research. It is argued in this paper that such considerations are not always considered, leaving important gaps in evidence-based decision-making, and creating, often unintentionally, male-centric services which do not meet the needs of female migrants. To remedy this, female migrant voices must be included in the design of mental health services. Access to specialist mental health support services within primary care needs to include assurances of fair treatment regardless of immigration status, for female migrants to have a greater chance of achieving better mental health outcomes. Treatment and support options may consider meanings and understanding of mental health and the importance spirituality holds for some individuals. When designing inclusive services, gender appropriateness should be a key consideration. Including definitions of terms such as ‘migrant’, ‘asylum seeker’ and ‘refugee’ may be helpful inclusions when considering the publication of research. Though migrant classifications are of great significance in identifying and understanding the varying needs of migrant populations, gender and sex similarities and differences and the intersectional nature of individuals and whole groups must be given similar priority to understand what the barriers and facilitators are to accessing mental health support in female migrant populations.

### Electronic supplementary material

Below is the link to the electronic supplementary material.

**Supplementary File 1**: Lived experience commentary on the Systematic Review.

**Supplementary File 2**: Initial Framework for the systematic review guided by Co-production Group Discussion.

## Data Availability

All data generated or analysed during this study are included in this published article [and its supplementary information files].
