# Supplementary material for: Barriers and facilitators to seeking and accessing mental health support in primary care and the community among female migrants in Europe: a “feminisms” systematic review
Source: Int J Equity Health. 2023 Sep 26;22:196. doi: 10.1186/s12939-023-01990-8 (PMC10523615; doi:10.1186/s12939-023-01990-8)
Supplement: Supplementary file 1 — Lived experience commentary on the Systematic Review. [file 12939_2023_1990_MOESM1_ESM.docx]

**Lived experience commentary on the Systematic Review**

Having reviewed the Results and Discussion sections of the Systematic Review, I reflect that there are many different barriers and facilitators for female migrants accessing support for mental health and that these barriers vary greatly depending on the culture, religion, and age of the female migrants.

There are many strengths of the studies including the following: the recruitment of co-researchers with lived migrant experiences; carrying out the data collection in community-based settings; the number of different countries in which the research was carried out and the many different countries from which the female migrants had originated. I particularly thought that Table 4 was an excellent tool for summarising the results of the research. I particularly found the sub-themes of stigma very informative.

For this review, I think it would have been informative to also include information on how the barriers and facilitators varied depending on the number of years the migrant had been in the country. I also think that other ways of accessing mental health apart from through the GP could have been reviewed.

I think that for any future research, there are groups of female migrants who should be recruited to participate. I think that female migrants who are currently isolated do not have much social contact and don’t currently have any community professionals involved in their lives should be recruited, as I am sure that their experience of the barriers to accessing mental health support would add useful information to the review.

JB
