# Supplementary material for: Barriers and facilitators to seeking and accessing mental health support in primary care and the community among female migrants in Europe: a “feminisms” systematic review
Source: Int J Equity Health. 2023 Sep 26;22:196. doi: 10.1186/s12939-023-01990-8 (PMC10523615; doi:10.1186/s12939-023-01990-8)
Supplement: Supplementary file 2 — Initial Framework for the systematic review guided by Co-production Group Discussion. [file 12939_2023_1990_MOESM2_ESM.docx]

Supplementary File 2: Initial Framework for the systematic review guided by Co-production Group Discussion

| **Initial themes** | **Initial categories discussed by the Coproduction group** | **Refined categories guided by literature review** | **Core concepts** | **Final themes for review** |
| --- | --- | --- | --- | --- |
| Barriers | - *We do not have enough information in our communities.* - *I didn’t know where to go for support. A lot of women I know do not know where to go.* | - Information Awareness - Lack of interpretation support - Services not providing easily accessible information - Services not aware of up-to-date information and guidance | - Access - Cultural - Structural - Individual level - Gender issues - Stigma | - Access to information - Cultural and spiritual barriers - Stigma - Structural barriers - Gender-specific barriers |
|  | - *We have difficulties discussing mental health in our communities* - *There is the issue of if I say something about my mental health I will be viewed as being crazy or something worse.* | - Self-stigma of MH. - The societal stigma of MH, including that of family members with MH ill-health. - Institutional stigma, including negative beliefs of reasons why migrants access MH services | - Individual - Cultural - Structural concepts of stigma - Stigma |  |
|  |  | - Service delivery does not consider gender. - Service delivery not supporting certain types of migrants. - Lack of interpretation services - ‘Hostile environment’ policies and practices | - Access - Cultural - Structural - Individual level - Gender issues - Stigma |  |
|  | *I think going to church helps a lot of the women I know. I always get help from the ladies in my bible study group.* | - The willingness of migrant females to discuss mental and spiritual health. - Spiritual leader awareness of mental health conditions - Supportive friends and family |  | Religiosity, community, and religious leaders |
| Facilitators | *We provide support for many migrants and most of our clients are females*  *We match female migrants with their preferred gender support.* | - Availability of culturally and gender-sensitive mental health support. - Peer support from other migrant females | Individual levels  Structural levels | Gender-sensitive support  Education settings  Resilience and adaptability explanations |
|  | *You must be strong in this world. I think I must work twice as hard as the Birish-born women. Now imagine how much harder I must work in a male-run world.* | - Resilience as a factor in the increased likelihood of seeking support. - Adaptability and acculturation to a new environment. |  |  |
|  |  |  |  |  |
